# Systematic approach for assessing whether undeletable chromosomal regions in *Saccharomyces cerevisiae* are required for cell viability

**DOI:** 10.1186/s13568-020-01001-x

**Published:** 2020-04-15

**Authors:** Naim Hassan, Farhana Easmin, Yu Sasano, Keisuke Ekino, Hisataka Taguchi, Satoshi Harashima

**Affiliations:** Department of Applied Microbial Technology, Faculty of Biotechnology and Life Science, Sojo University, Ikeda 4-22-1, Nishi-ku, Kumamoto, 860-0082 Japan

**Keywords:** Undeletable regions, Synthetic lethality, Mini-chromosome loss, Yeast

## Abstract

Previously, we identified 49 undeletable chromosomal regions harboring only non-essential genes in the genome of *Saccharomyces cerevisiae*. We proposed that there might be unknown synthetic lethal combinations of genes present in such undeletable regions of the genome. In this study, we chose four of the smallest undeletable chromosomal regions among the 49 and performed extensive further analyses to narrow down the gene-pairs responsible for lethality by replacing sub-regions in various combinations with a DNA module comprising the *CgLEU2* marker. Although the methodology was different from previous study, interestingly the results revealed that not only the sub-regions but also the entire region was replaceable. To solve the apparent discrepancy between previous and present results, we further conducted additional analysis including investigation of suppressor mutation and mini-chromosome loss assay through the construction of mini-chromosome harboring two particular chromosomal regions with marked with *URA3* marker by employing 5-FOA system. Based upon careful observation on the phenotype of colony formation on 5-FOA medium by spot test, we came to an important conclusion that particular chromosomal regions harboring only non-essential genes can be categorized into three classes, i.e., essential, non-essential and intrinsically essential. Intrinsically essential region is defined as appearance of papillae after mini-chromosome loss which implicates that the region is essential but compensatable against cell lethality. Our present study indicates that prudent and multiple approaches as performed in this study are needed to judge whether a particular chromosomal region of the *S. cerevisiae* genome is essential, non-essential or intrinsically essential but compensatable.

## Introduction

Discovering genetic interaction networks is required for identifying novel genes and pathways and for predicting similar networks in genomes. Baker’s yeast *Saccharomyces cerevisiae* is largely used and best characterized single-celled eukaryotic model for the study of a variety of biological processes (Karathia et al. 2011). More than 80% of the genes in *S. cerevisiae* are not required for cell proliferation in nutrient medium. This makes *S. cerevisiae* a useful experimental organism to reveal the function of non-essential genes (Winzeler et al. 1999; Giaever et al. 2002). The inactivation of some non-essential genes in specific combinations can have a lethal effect (Novick et al. 1989; Guarente 1993). This property makes the yeast genome resistant to engineering and could be problematic for generating new strains. Synthetic lethal genetic interactions have been extensively studied in *S. cerevisiae* using synthetic genetic array (SGA) analysis, in which a query mutation is systematically crossed with almost all viable deletion mutants to obtain double-mutant meiotic progeny (Tong et al. 2001, 2004; Giaever et al. 2002). However, formation of double mutants in SGA analysis depends on meiotic recombination. Double mutant construction is not possible if the two genes to be combined are tightly linked on the same chromosome. As a consequence, numerous linked gene-pairs that form small colonies of double mutants have been overlooked in SGA (Kaboli et al. 2014).

To overcome the limitation of constructing double mutants of two tightly linked genes on the same chromosome, we developed PCR-mediated chromosome deletion technology (PCD). Deletion of all regions harboring only non-essential genes throughout the genome led to the interesting discovery that 49 chromosomal regions were undeletable (Kaboli et al. 2014). This result indicates that there might be unknown lethal combinations of non-essential genes present in these 49 undeletable regions, which were not reported by SGA. This finding motivated us to identify the genes responsible for the synthetic lethality in all of the 49 undeletable regions. In this study, we chose four of the smallest undeletable regions from these 49 regions and attempted to narrow down the genes responsible for lethality by replacing the sub-regions with DNA modules harboring markers in various combinations.

## Materials and methods

### Strains, plasmids and media

The strains and the plasmids used in this study are listed in Table 1. *S. cerevisiae* strain SJY4 was used as a parental strain for the replacement of chromosomal regions. The strain SJY576, where the Chr2-6 (Chromosome 2, 318749–330960) region was replaced by a DNA module harboring *CgLEU2*, was used as a host strain for splitting the left edge of *CgLEU2*. The strain SJY577, transformants of SJY576 where the left edge of *CgLEU2* was split, was used as a host strain for splitting the right edge of *CgLEU2*. Some of the transformants constructed in previous study (Kaboli et al. 2014) and transformants constructed in present study were used for spot test. We used the loxP site-deleted plasmid pSJ69 (Easmin et al. 2019a) and pSJ70 (Easmin et al. 2019b) derived from p3008 and p3009, respectively (Sugiyama et al. 2005) as templates in which loxP-flanked DNA sequences were deleted to avoid undesired site-specific recombination. The plasmid pSJ69 harboring selective marker *Candida glabrata LEU2* (*CgLEU2*) was used as a template to synthesize a DNA module for replacement of a particular chromosomal region. The plasmid pSJ70 harboring *CgHIS3*, loxP site-deleted plasmid pSJ23 (Easmin et al. 2019b) harboring a *URA3* marker and the plasmid p3121 (Sugiyama et al. 2005) harboring *CEN4* were used to split the left and right edge of the DNA module-replaced chromosomal region. The plasmids p3121 and pSJ23 were used to construct DNA modules to duplicate target chromosomal regions.

**Table 1:** Strains and plasmids used in this study

Strain or plasmid	Description	Remarks/references
Strains		
SJY4	*MATα his3Δ1 leu2Δ0 lys2Δ0 ura3Δ0*	Winston et al. (1995)
SJY576	Leu^+^ Transformant of SJY4, Chr2-6 region replaced with *CgLEU2*	This study
SJY577	His^+^ Transformants of SJY576 for left edge splitting of *CgLEU2* module with *CgHIS3*	This study
SH30072	Ura^+^ Transformant of SJY4, harboring Chr1-2 region in the mini-chromosome	Kaboli et al. (2014)
SH30075	Ura^+^ Transformant of SJY4, harboring Chr2-2 region in the mini-chromosome	Kaboli et al. (2014)
SH30077	Ura^+^ Transformant of SJY4, harboring Chr2-4 region in the mini-chromosome	Kaboli et al. (2014)
SH30079	Ura^+^ Transformant of SJY4, harboring Chr2-6 region in the mini-chromosome	Kaboli et al. (2014)
SH30080	Ura^+^ Transformant of SJY4, harboring Chr2-7 region in the mini-chromosome	Kaboli et al. (2014)
SH30084	Ura^+^ Transformant of SJY4, harboring Chr3-2 region in the mini-chromosome	Kaboli et al. (2014)
Plasmids		
pSJ23	A derivative of pUG6 carrying *URA3*	Easmin et al. (2019b)
pSJ69	loxP site-deleted p3008	Easmin et al. (2019a)
pSJ70	loxP site deleted p3009	Easmin et al. (2019b)
p3121	The *CEN4* module containing plasmid constructed by modifying pUG6	Sugiyama et al. (2005)

Yeast strains were grown at 30 °C in YPDA medium (2% peptone, 2% glucose, 1% yeast extract and 0.004% adenine HCL). Supplemented minimal medium (SMM) (0.67% yeast nitrogen base without amino acids [Difco, Sparks, MD, USA], 20 mg/L to 100 mg/L amino acids [l-Leucine, l-Histidine, l-Lysine HCL, l-Methionine and l-Tryptophan] and nucleic acid bases [Adenine HCL, Uracil] and 2% glucose) lacking specific amino acids or nucleic acid bases were used to select transformants to examine auxotrophic phenotypes. 5-Fluoroorotic acid (5-FOA) medium, prepared according to Kaboli et al. (2014), was used to screen clones for the presence of the *URA3* marker gene. For plate assays, agar (2% w/v) was added to solidify the medium.

### Preparation of DNA modules

**Fig. 1:**
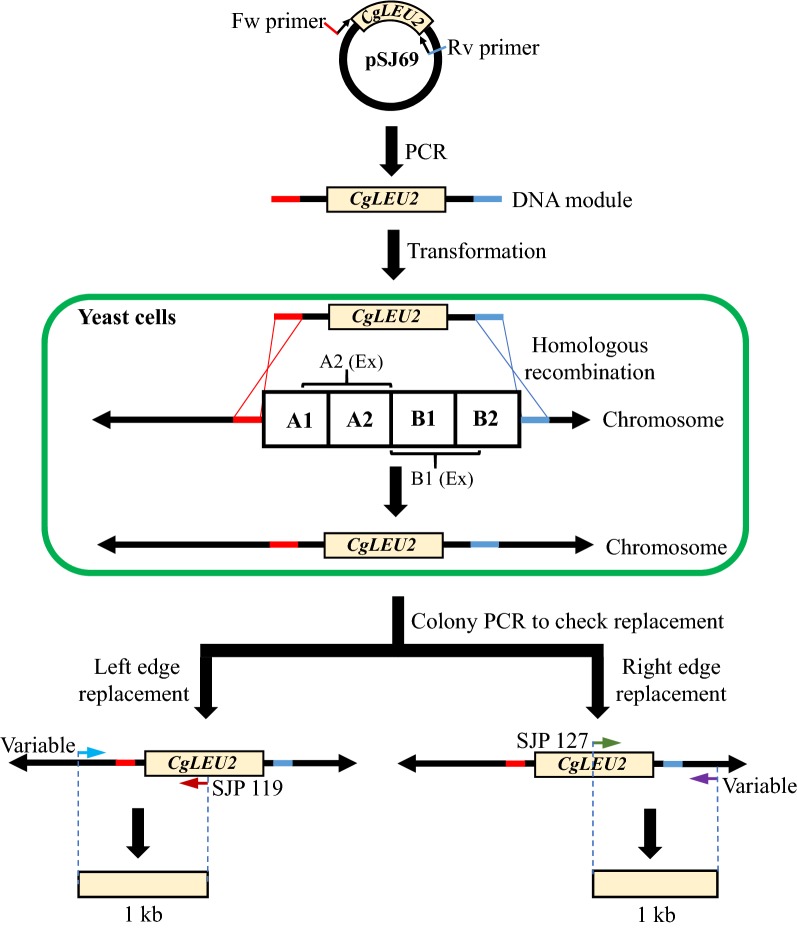
Overview of replacement analysis of target region. Target region was replaced by DNA module harboring *CgLEU2*. For amplification of DNA module, forward and reverse primers were designed to anneal with the plasmid pSJ69 and DNA module was amplified by PCR. DNA module has 50 bp homology sequence with the target region (harboring A1, A2, A2 (Ex), B1, B1 (Ex) and B2 sub-regions) in both edges. After introduction of DNA module by transformation into yeast cell, target region was supposed to be replaced by the DNA module through homologous recombination. Replacement of left edge of target region was checked by colony PCR with specific forward primers depending upon the target regions and common reverse primer SJP 119 which leads to PCR product of 1 kb. On the other hand, replacement of right edge of target region was checked with common forward primer SJP 127 and specific reverse primers depending upon respective regions which also amplified 1 kb band

Several types of DNA module to replace, split or duplicate target regions were prepared by PCR. To construct DNA modules for replacing target regions, the forward primer was designed by choosing a 50 bp sequence just prior to the target region using the Saccharomyces Genome Database (SGD: http://www.yeastgenome.org) and an additional 20 bp sequence homologous to the 5′-GGCCGCCAGCTGAAGCTTCG-3′ sequence of plasmid pSJ69. Likewise, the reverse primer was also designed by choosing a 50 bp reverse sequence just after the respective target region using SGD and an additional 20 bp reverse sequence homologous to the 5′-AGGCCACTAGTGGATCTGAT-3′ sequence of plasmid pSJ69 (Fig. 1). Splitting modules were prepared according to Sasano et al. (2016) by using pSJ70, pSJ23 and p3121 as template plasmids. The duplication module was prepared according to Natesuntorn et al. (2015) with slight modification. Specifically, rather than a 400 bp homology region used by Natesuntorn et al. (2015), we used a 50 bp homology sequence to duplicate the target regions. Primers used for making DNA modules for replacement, splitting or duplicating target regions are listed in Additional file 1: Table S1.

### Yeast transformation, colony PCR

Yeast cells were transformed according to the method of Gietz and Schiestl (2007). For selection of yeast transformants, cells were plated on appropriate selection medium.

Colony PCR and subsequent agarose gel electrophoresis were performed to check whether the expected replacement, splitting or duplication of the target chromosomal region had occurred in the transformants. Colony PCR was conducted according to Easmin et al. (2019a). Primers for colony PCR used to check replacement, splitting and duplication are listed in Additional file 1: Table S2.

### Mini-chromosome loss assay and spot test

Transformants to be tested were cultivated overnight in YPDA liquid medium and after serial dilution, cells were plated on YPDA plates (master plate) and incubated at 30 °C for 48 h. Colonies were replica-plated on SMM plates without uracil (Ura minus), without leucine (Leu minus), without leucine and uracil (Leu minus and Ura minus), 5-FOA and fresh YPDA plates and incubated at 30 °C for 24 to 72 h. Spot test was performed according to Kaboli et al. (2014).

## Results

### Identification of non-essential genes responsible for synthetic lethality of four undeletable chromosomal regions

**Fig. 2:**
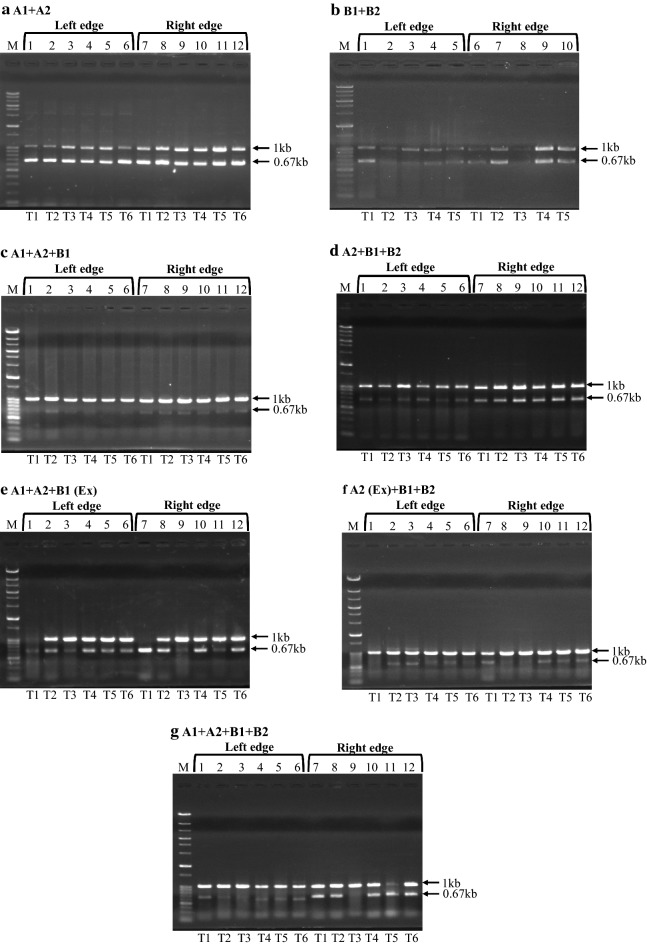
Colony PCR analysis of replaced sub-regions of Chr2-6 region. Each lane represents checking of left or right edge replacement of Chr2-6 sub-regions in independent transformants (T1, T2, T3, T4, T5 and T6). M represents size marker (Gene Ladder Wide 2, Nippon Gene, Toyama, Japan). A common set of primers (SJP 121 and SJP 242) was used in all PCR verification experiments to amplify the 0.67 kb *CNE1* gene on Chromosome 1 as an internal control. 1 kb band was the expected band for replacement of either left or right edge of sub-regions. **a** SJP 118 and SJP 119 were used for checking left edge whereas SJP 127 and SJP 384 were used for checking right edge replacement of A1 + A2 sub-regions, respectively. **b** SJP 390 and SJP 119 were used for checking left edge whereas SJP 127 and SJP 385 were used for checking right edge replacement of B1 + B2 sub-regions, respectively. **c** SJP 118 and SJP 119 were used for checking left edge whereas SJP 127 and SJP 457 were used for checking right edge replacement of A1 + A2 + B1 sub-regions, respectively. **d** SJP 427 and SJP 119 were used for checking left edge whereas SJP 127 and SJP 385 were used for checking right edge replacement of A2 + B1 + B2 sub-regions, respectively. **e** SJP 118 and SJP 119 were used for checking left edge whereas SJP 127 and SJP 479 were used for checking right edge replacement of A1 + A2 + B1 (Ex) sub-regions, respectively. **f** SJP 483 and SJP 119 were used for checking left edge whereas SJP 127 and SJP 385 were used for checking right edge replacement of A2 (Ex) + B1 + B2 sub-regions, respectively. **g** SJP 118 and SJP 119 were used for checking left edge whereas SJP 127 and SJP 385 were used for checking right edge replacement of entire Chr2-6 region, respectively

According to our previous study (Kaboli et al. 2014), 49 chromosomal regions containing only non-essential genes were identified to be undeletable from the *S. cerevisiae* genome. These observations indicate that yeast cells cannot survive if these regions are deleted and further suggests that the regions are likely to harbor genes responsible for synthetic lethality. To identify genes responsible for the synthetic lethality, we embarked on a systematic study of all 49 regions with the aim of pinpointing gene-pairs in the respective undeletable regions. As a part of this comprehensive project, we selected four of the smallest chromosomal regions, i.e., Chr2-6 (Chromosome 2: 318749–330960; 12.2 kb), Chr9-2 (Chromosome 9: 87850–102249; 14.4 kb), Chr2-2 (Chromosome 2: 21866–37346; 15.5 kb) and Chr11-2 (Chromosome 11: 188434–204755; 16.3 kb) (SGD: http://www.yeastgenome.org). Here, we employed an approach to narrow down the regions responsible for synthetic lethality by using genome engineering technology. For this purpose, we divided each region into 6 sub-regions called A1, A2, A2 Extension (Ex), B1, B1 Extension (Ex) and B2 (Fig. 1). We then attempted to delete these sub-regions through replacement of DNA modules in various combinations for all regions. The combinations that we tested were A1 + A2, B1 + B2, A1 + A2 + B1, A2 + B1 + B2, A1 + A2 + B1 (Ex) and A2 (Ex) + B1 + B2. Results of the replacement by transformation experiments of these regions are shown in Table 2. If replacement of a particular sub-region results in lethality, transformants should not be obtained. By contrast, if deletion of the same sub-region does not lead to lethality, viable transformants will be obtained. In all cases a substantial number of viable transformants were isolated (Table 2). Transformants were verified by randomly picking two to six of them and performing structural analysis of the chromosomes by colony PCR. In colony PCR, the replacement of the left and right edge of the respective chromosomal sub-regions were checked (Fig. 2a–f; Additional file 2: Figs. S1–S3). Results of colony PCR revealed that most of the transformants had the expected chromosomal structure, indicating that each targeted chromosomal sub-region was replaced by a DNA module harboring *CgLEU2* (Table 2). From these observations, we concluded that none of the sub-regions harbor genes responsible for synthetic lethality.

**Table 2:** Replacement of various chromosomal regions

Name of the region (co-ordinate number)	Replaced sub regions (co-ordinate number)	Number of transformants	Number of transformants analyzed	Transformants with expected replacement	Replaceable/non-replaceable
Chr2-2 (21866–37346)	A1 + A2 (21866–29606)	771	6	6	Replaceable
	B1 + B2 (29606–37346)	1001	6	5	Replaceable
	A1 + A2 + B1 (21866–33476)	200	6	4	Replaceable
	A2 + B1 + B2 (25737–37346)	611	6	6	Replaceable
	A1 + A2 + B1 (Ex) (21866–36036)	314	6	4	Replaceable
	A2 (Ex) + B1 + B2 (24473–37346)	289	6	6	Replaceable
	A1 + A2 + B1 + B2 (21866–37346)	494	6	4	Replaceable
Chr2-6 (318749–330960)	A1 + A2 (318749–324853)	930	6	6	Replaceable
	B1 + B2 (324856–330960)	1171	5	5	Replaceable
	A1 + A2 + B1 (318749–327908)	411	6	6	Replaceable
	A2 + B1 + B2 (321802–330960)	626	6	6	Replaceable
	A1 + A2 + B1 (Ex) (318749–329133)	316	6	5	Replaceable
	A2 (Ex) + B1 + B2 (321064–330960)	256	6	6	Replaceable
	A1 + A2 + B1 + B2 (318749–330960)	769	6	6	Replaceable
Chr9-2 (87850–102249)	A1 + A2 (87850–95050)	731	5	5	Replaceable
	B1 + B2 (95050–102249)	703	2	1	Replaceable
	A1 + A2 + B1 (87850–98650)	400	6	5	Replaceable
	A2 + B1 + B2 (91451–102249)	452	6	3	Replaceable
	A1 + A2 + B1 (Ex) (87850–100181)	313	6	6	Replaceable
	A2 (Ex) + B1 + B2 (89524–102249)	211	6	6	Replaceable
	A1 + A2 + B1 + B2 (87850–102249)	445	6	3	Replaceable
Chr11-2 (188434–204755)	A1 + A2 (188434–196595)	922	6	6	Replaceable
	B1 + B2 (196595–204755)	965	6	6	Replaceable
	A1 + A2 + B1 (188434–200675)	443	6	2	Replaceable
	A2 + B1 + B2 (192515–204755)	627	6	6	Replaceable
	A1 + A2 + B1 (Ex) (188434–201328)	364	6	6	Replaceable
	A2 (Ex) + B1 + B2 (190334–204755)	217	6	4	Replaceable
	A1 + A2 + B1 + B2 (188434–204755)	515	6	4	Replaceable

**Fig. 3:**
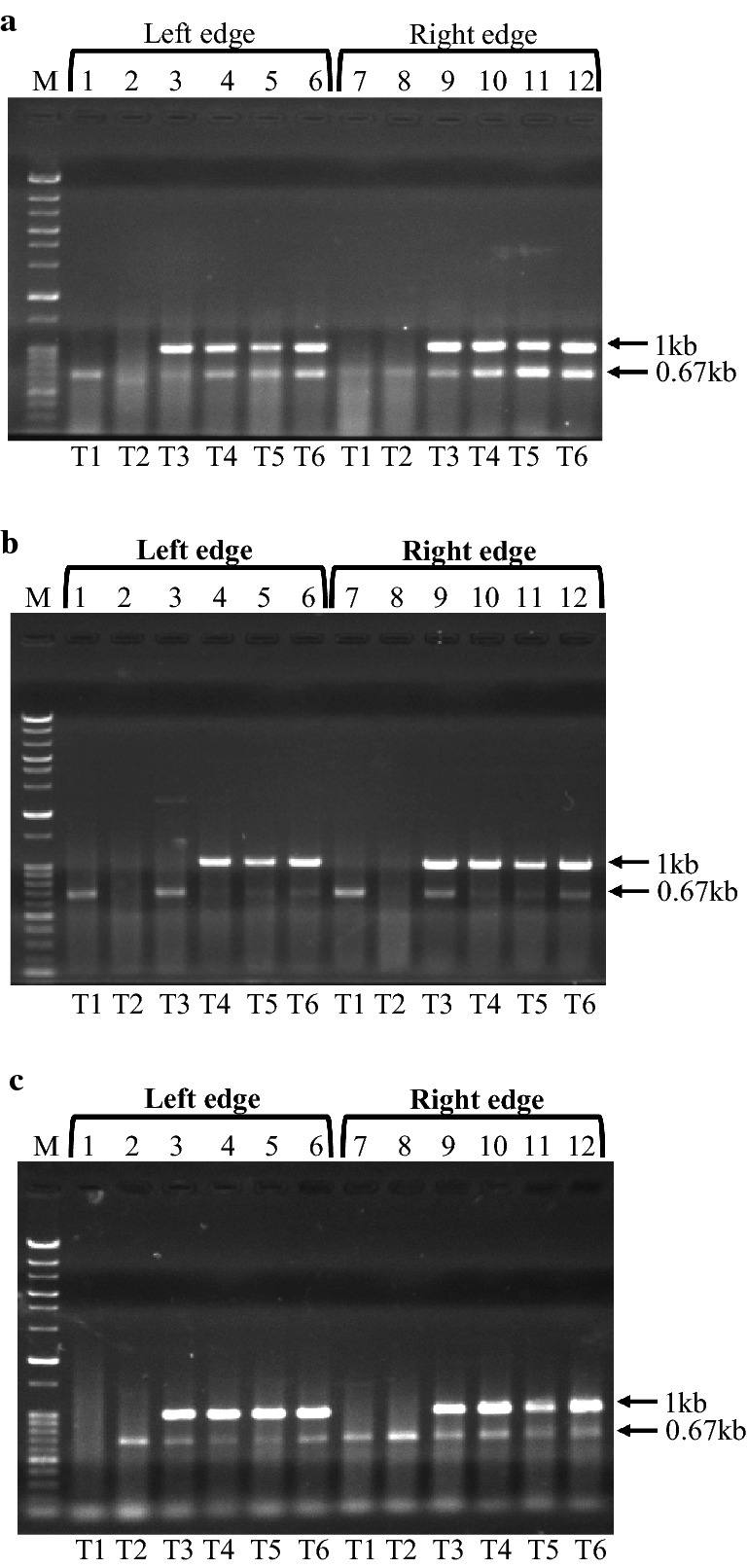
Colony PCR analysis of replaced Chr2-2, Chr9-2 and Chr11-2 regions. Each lane represents checking of left or right edge replacement of entire Chr2-2, Chr9-2 and Chr11-2 regions in independent transformants (T1, T2, T3, T4, T5 and T6), respectively. 1 kb band was the expected band for replacement of either left or right edge of entire regions. **a** SJP 217 and SJP 119 were used for checking left edge whereas SJP 127 and SJP 383 were used for checking right edge replacement of Chr2-2 region, respectively. **b** SJP 215 and SJP 119 were used for checking left edge whereas SJP 127 and SJP 369 were used for checking right edge replacement of Chr9-2 region, respectively. **c** SJP 219 and SJP 119 were used for checking left edge whereas SJP 127 and SJP 387 were used for checking right edge replacement of Chr11-2 region, respectively

These observations motivated us to check whether the whole region could be replaced by the *CgLEU2* marker. First, transformation experiments were performed to replace the entire Chr2-6 region. In this experiment, we obtained 769 transformants for the replacement of the Chr2-6 region (Table 2) and subsequent analysis by colony PCR showed that six out of six transformants had the expected structural alteration (Fig. 2g), indicating that the entire Chr2-6 region could be replaced with *CgLEU2*. We also conducted a similar experiment for the other three chromosomal regions, Chr2-2, Chr9-2 and Chr11-2. These studies showed the other three chromosomal regions could also be replaced by the *CgLEU2* marker without causing lethality (Fig. 3). These results are inconsistent with our previous findings (Kaboli et al. 2014), which showed that these regions cannot be deleted. However, the methodology in the previous work was different. Thus, we performed additional experiments described in the next section to explore the apparent inconsistency.

### Transformants harboring a mini-chromosome comprising only genetic markers are viable

We noted following difference in methodology between this study and the previous study might explain the apparently contradictory results. In the previous study, Kaboli et al. (2014) constructed a mini-chromosome comprising target regions marked with the *CgURA3* gene by PCR-mediated one-step splitting (PCS) technology. Mini-chromosome loss assays were then performed to analyze whether a particular region was essential for cell viability. In all, 49 regions were found to be undeletable. This conclusion was based upon the observation that they did not see colony formation by transformants harboring the mini-chromosome on 5-FOA medium. By contrast, in this study, deletion through replacement of four out of the 49 regions with a DNA module did not result in lethality. To investigate why deletion through replacement of these chromosomal regions rather than simple deletion gave us viable transformants, we split the left and right edge of one of the replaced regions (Chr2-6) by PCS technology (Sasano et al. 2016). We then constructed a mini-chromosome consisting of *CgLEU2* and *URA3* marker for performing mini-chromosome loss assays by the 5-FOA method.

**Fig. 4:**
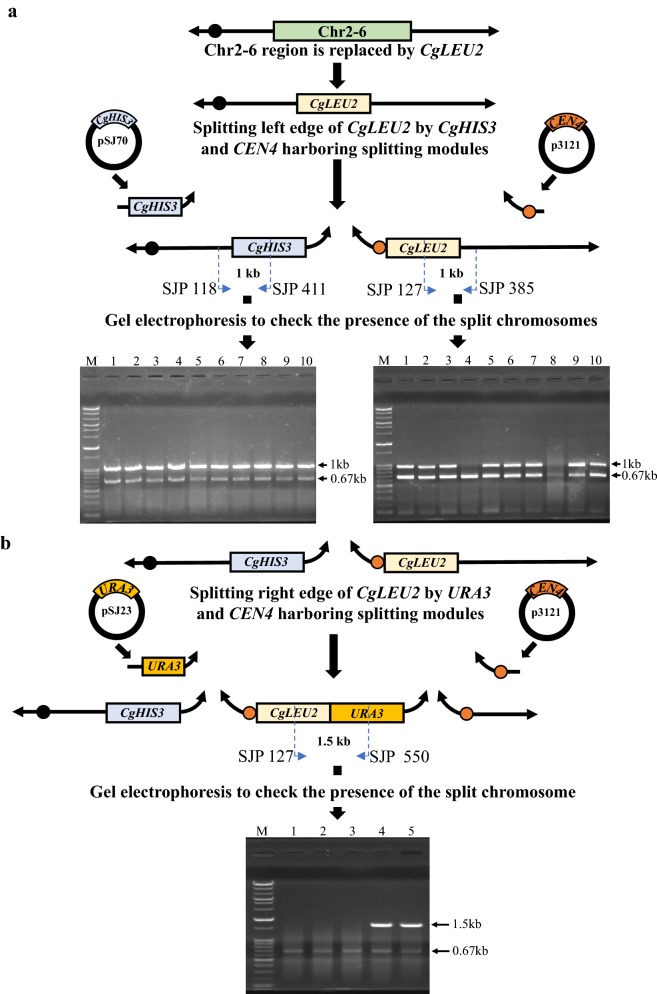
Sequential splitting left and right edge of Chr2-6 replaced transformants. **a** Two splitting modules (One module synthesized from the plasmid pSJ70 contained *CgHIS3* and the other module synthesized from the plasmid p3121 contained *CEN4* as a centromere) were introduced into the host strain SJY 576 (Chr2-6 region replaced transformants) to split left edge of *CgLEU2.* Bottom part of **a** represents gel electrophoresis of colony PCR and Lane 1 to 10 represents 10 independent transformants were checked to amplify 1 kb band denoting the left edge of *CgHIS3* and right edge of *CgLEU2*. Primers used for colony PCR are illustrated in **a**. **b** After splitting left edge of *CgLEU2* in Chr2-6 region replaced transformants (SJY 577), we tried to split sequentially the right edge of *CgLEU2*. 2 splitting modules (One module synthesized from the plasmid pSJ23 contained *URA3* and another module synthesized from the plasmid p3121 contain *CEN4* as a centromere) were introduced into SJY 577 to split right edge of *CgLEU2*. Bottom part of **b** represents gel electrophoresis of colony PCR and 5 independent transformants were checked for amplifying 1.5 kb band denoting the right edge of *CgLEU2* and left edge of *URA3*. Primers used for colony PCR are illustrated in **b**

For generating a mini-chromosome, we first split (between nucleotide no. 318748 of Chromosome 2 and nucleotide no. 1 of *CgLEU2* sequence) the left edge of the Chr2-6 region, which had been replaced by a DNA module containing the *CgLEU2* marker. This experiment was done by using two kinds of splitting module, one of which contained the *CgHIS3* marker and the other of contained *CEN4* (Fig. 4a). Transformants were selected on SMM medium without leucine and histidine. In all, 827 Leu^+^ His^+^ transformants were obtained (Table 3), ten of which were arbitrarily picked for analysis by colony PCR. Eight of the ten transformants had the anticipated splitting at the left edge of the Chr2-6 replaced region (Fig. 4a). Among these eight transformants, one (called SJY577) was selected for subsequent splitting (between nucleotide no. 1685 of *CgLEU2* sequence and nucleotide no. 330961 of Chromosome 2) at the right edge of the *CgLEU2* marker of a newly generated split chromosome. In this transformation experiment, we used two splitting DNA modules; one module contained the *URA3* marker and the other module contained *CEN4* (Fig. 4b). We selected transformants on SMM medium without leucine, histidine and uracil. Five out of 917 Leu^+^ His^+^ Ura^+^ transformants obtained (Table 3) were arbitrarily picked and checked by colony PCR. Two out of five transformants had the expected splitting at the right edge of the Chr2-6 replaced region (Fig. 4b). In this way, Chromosome 2 was split into three parts to generate a mini-chromosome comprising only *CgLEU2* (DNA module) and the *URA3* marker. These cells, like those harboring unsplit Chromosome 2, were viable despite the entire Chr2-6 chromosomal region being deleted from the genome.

**Table 3:** Splitting left and right edge of replaced Chr2-6 region

Region replaced by *CgLEU2*	Splitting point	Number of transformants	Number of transformants analyzed	Transformants with expected splitting
Chr2-6	Left edge	827	10	8
	Right edge of replaced Chr2-6 left split transformant	917	5	2

### Assessing whether the newly generated mini-chromosome is essential for cell viability

**Fig. 5:**
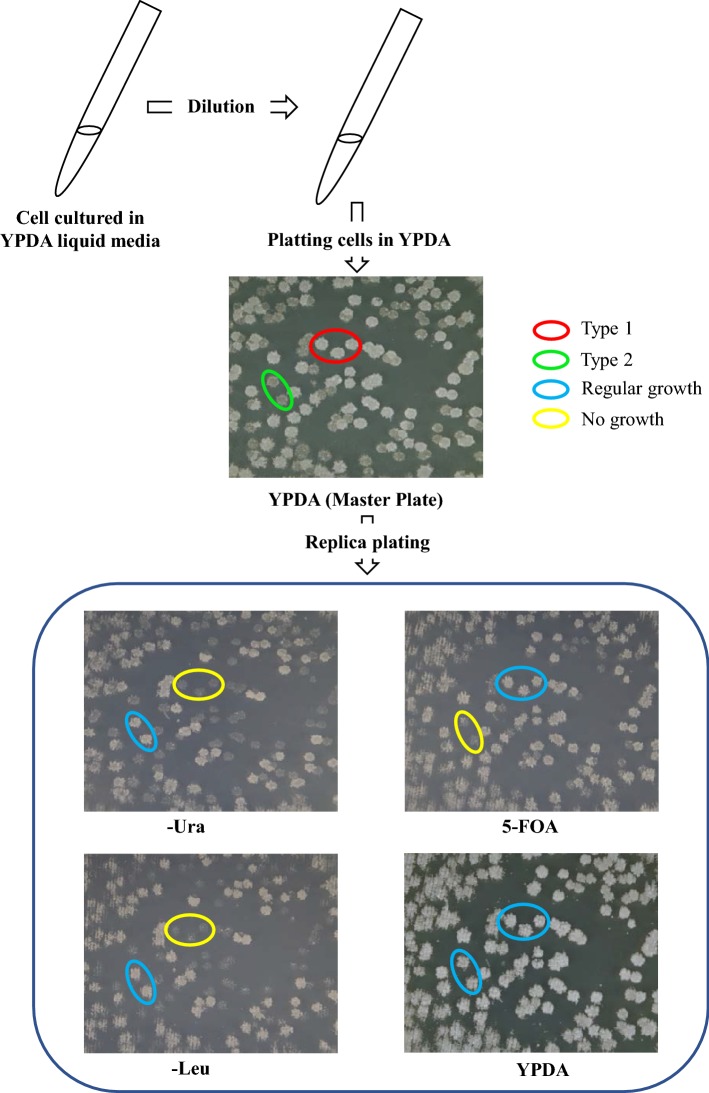
Mini-chromosome loss assay. Transformants (constructed by splitting left and right edge of *CgLEU2* replaced Chr2-6 region) harboring mini-chromosome consisting of only *CgLEU2* and *URA3* markers were cultured in liquid YPDA medium and subjected to dispense on YPDA plate after appropriate dilution. After colony formation, this plate was used as a master plate to replica plating on Ura minus, Leu minus, 5-FOA and YPDA media (as a control). After replica plating, two types of colonies were appeared in YPDA master plate, we named them as Type 1 and Type 2. To distinguish them, three Type 1 colonies were circled by red color and two Type 2 colonies were circled by green color. In other replica-plated plates, colonies that showed regular growth were circled by blue color and colonies that showed no growth were circled by yellow color. Type 1 colonies could not grow on Ura minus, Leu minus media but could grow on 5-FOA and YPDA media. On the other hand, Type 2 colonies could grow on Ura minus, Leu minus and YPDA media but could not grow on 5-FOA medium

Cells harboring a mini-chromosome were cultivated in liquid YPDA medium, plated on YPDA plate (treated as a master plate for replica plating) and replica-plated on Ura minus, Leu minus, and 5-FOA along with YPDA media (as a control) and incubated for 24 h (Fig. 5). Two kinds of colonies were observed on the YPDA master plate (Fig. 5). One type of colony (Type 1) showed growth on 5-FOA and YPDA control media but no growth on Ura minus and Leu minus media (i.e. Ura^_^ and Leu^_^ colonies). The second type of colony (Type 2) showed growth on Ura minus and Leu minus media and YPDA control plates (i.e. Ura^+^ and Leu^+^ colonies) but no growth on 5-FOA medium. Because Ura^−^ (and Leu^−^) cells are considered to have lost the mini-chromosome, growth of these cells on YPDA and 5-FOA medium indicates that the mini-chromosome is not required for viability. This result confirmed the findings described in the previous section. However, there remains an apparent inconsistency with the results obtained by Kaboli et al. (2014), which showed that loss of the Chr2-6 region was lethal to the cells. We reasoned that there might be an unknown suppressor mutation somewhere in the 16 chromosomes that suppresses lethality. Indeed, there is intrinsic selection pressure which may induce suppressor mutations that suppress lethality caused by deletion of an essential region of the chromosome. To explore this hypothesis, we performed further experiments described in the next section.

### Checking the suppressor mutation hypothesis

**Fig. 6:**
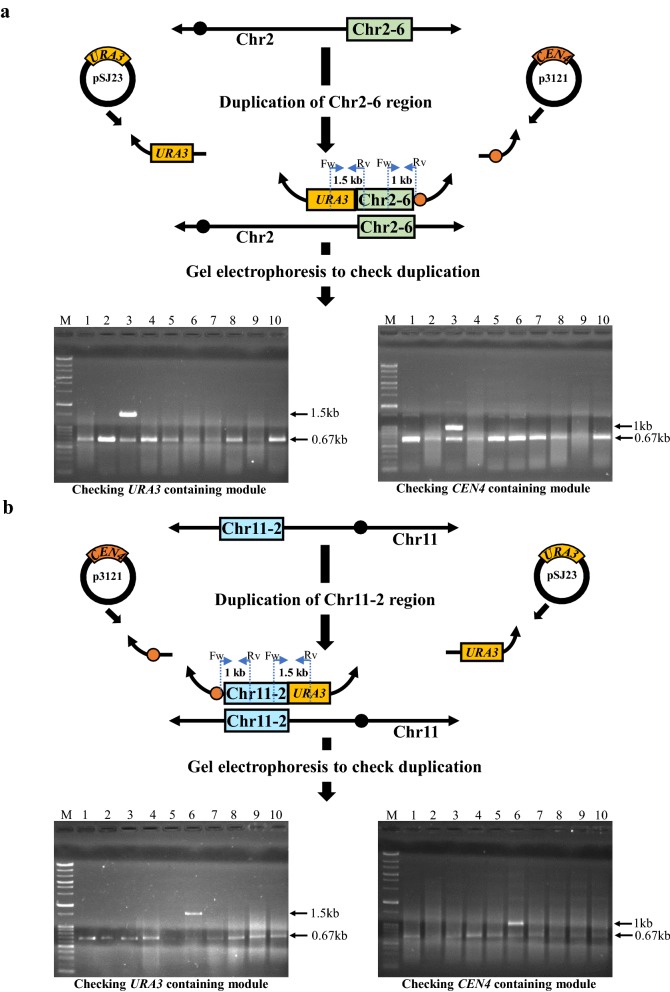
Duplication of Chr2-6 and Chr11-2 regions and checking transformants by colony PCR. Upper part of **a**, **b** represents 2 duplication modules (One module synthesized from the plasmid pSJ23 contained *URA3* and another module synthesized from the plasmid p3121 contained *CEN4* as a centromere) were introduced into SJY4 to duplicate Chr2-6 and Chr11-2 regions, separately. Bottom part of **a**, **b** represents gel electrophoresis of colony PCR and each lane represents independent transformants without the most left lane, which contains size marker. In **a**, SJP 550 and SJP 690 were used to check *URA3* harboring DNA module whereas SJP 694 and SJP 697 were used to check *CEN4* harboring DNA module for the duplication of Chr2-6 region, respectively. In **b**, SJP 692 and SJP 550 were used to check *URA3* harboring DNA module whereas SJP 697 and SJP 696 were used to check *CEN4* harboring DNA module for the duplication of Chr11-2 region, respectively

**Fig. 7:**
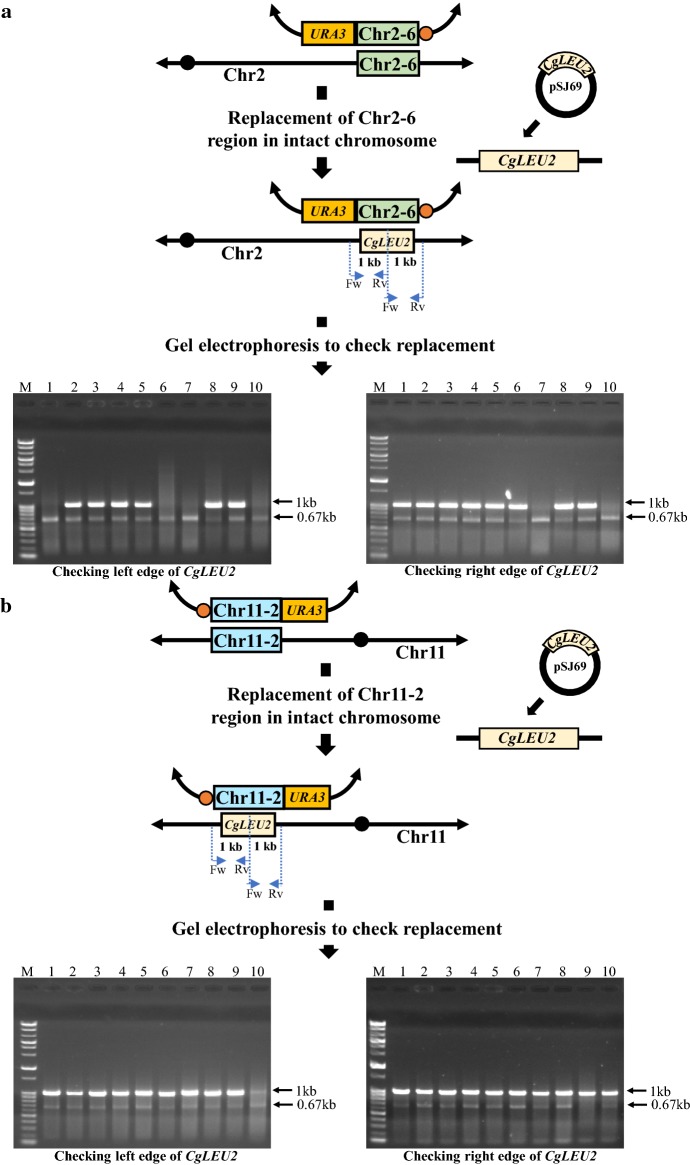
Checking Chr2-6 (dup + rep) and Chr11-2 (dup + rep) transformants. Upper part of **a**, **b** represents DNA module (synthesized from the plasmid pSJ69 as template contained *CgLEU2*) was introduced into Chr2-6 and Chr11-2 duplicated transformants to replace Chr2-6 and Chr11-2 regions from the intact chromosome, respectively. Bottom part of **a**, **b** represents gel electrophoresis of colony PCR and each lane represents independent transformants without the most left lane, which contains size marker. In **a**, SJP 118 and SJP 119 were used to check left edge of *CgLEU2* whereas SJP 127 and SJP 385 were used to check right edge of *CgLEU2* for the replacement of Chr2-6 region in Chr2-6 duplicated transformants, respectively. In **b**, SJP 219 and SJP 119 were used to check left edge of *CgLEU2* whereas SJP 127 and SJP 387 were used to check right edge of *CgLEU2* for the replacement of Chr11-2 region in Chr11-2 duplicated transformants, respectively

To investigate the suppressor mutation hypothesis, we arbitrarily chose the Chr2-6 and Chr11-2 regions and duplicated each region using a DNA module harboring *URA3* and *CEN4* in strain SJY4 by PCDup technology. These experiments generated transformants harboring either the Chr2-6 or Chr11-2 regions on a mini-chromosome. In all, 1208 and 892 transformants were obtained for duplication of the Chr2-6 and Chr11-2 regions, respectively. Ten transformants from the two separate experiments were picked at random (Fig. 6a, b). In each case, one of the ten transformants had the expected duplication. Next, the Chr2-6 or Chr11-2 region were replaced from the intact chromosome by a DNA module harboring *CgLEU2*. In all, 130 and 33 transformants were obtained for the replacement of the Chr2-6 and Chr11-2 regions from the Chr2-6 and Chr11-2 duplicated transformants, respectively. Ten transformants from each experiment were subsequently picked at random and analyzed. Six and ten transformants were found to have the expected structure for the replacement of Chr2-6 and Chr11-2 regions on intact chromosome in Chr2-6 and Chr11-2 duplicated transformants, respectively (Fig. 7a, b). We named these transformants Chr2-6 (dup + rep) and Chr11-2 (dup + rep) and subsequently performed mini-chromosome loss assays.

**Fig. 8:**
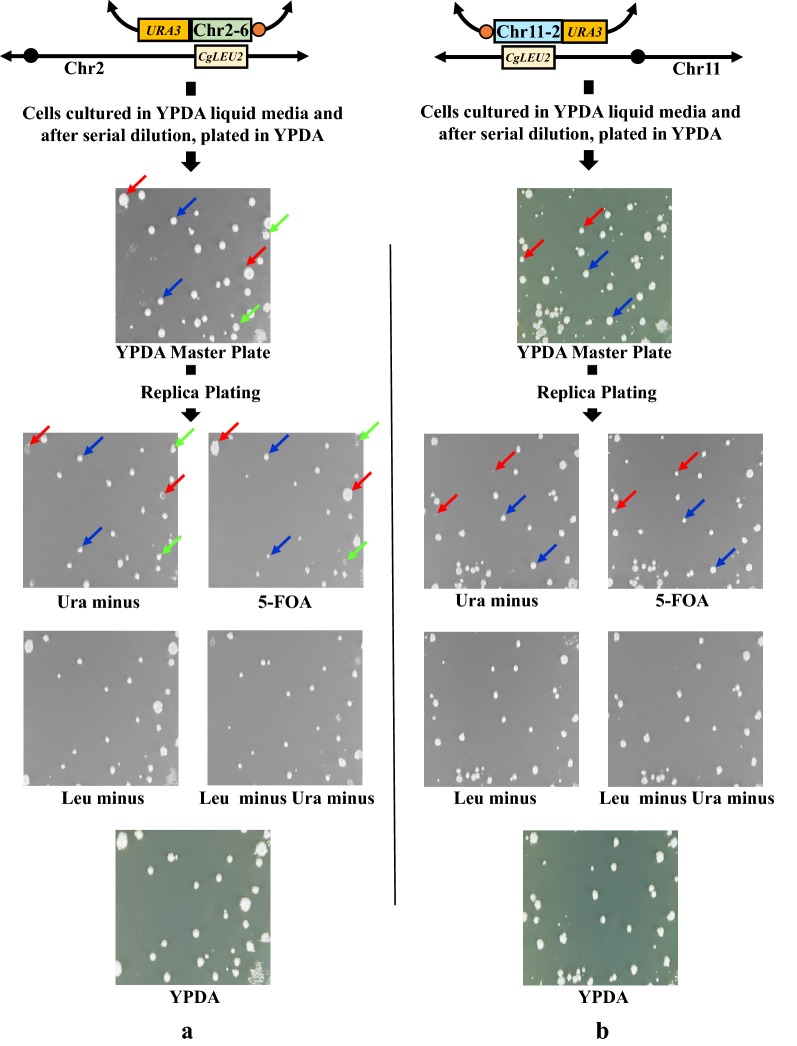
Mini-chromosome loss assay of duplicated mini-chromosome. **a** Represents transformants harboring Chr2-6 (dup + rep) and **b** represents transformant containing Chr11-2 (dup + rep), respectively. After serial dilution of full growth culture in YPDA liquid medium, cells were plated on YPDA plate. This YPDA plate was treated as a master plate for subsequent replica-plating experiment. After colonies appeared on YPDA plate, the YPDA plate was replica-plated on Leu minus, Ura minus, Leu and Ura minus media, 5-FOA and YPDA plates as a control. After incubation, we found that cells plated on YPDA master plate formed three types of colonies for Chr2-6 (dup + rep) and two types of colonies for Chr11-2 (dup + rep) cases. In both cases, one type of colonies were supposed to lose mini-chromosome. As a consequence, they showed Ura^−^ and 5-FOA^+^ phenotype. Two representatives of this type of colonies were marked with red-colored arrow. On the other hand, most of other colonies of both cases were supposed to keep mini-chromosome for which they showed Ura^+^ phenotype but they also formed colonies in 5-FOA medium. We think that these colonies consist of cells lost mini-chromosome and therefore they showed 5-FOA^+^ phenotype. Two of this type of colonies were marked with blue-colored arrow. Besides, in only Chr2-6 (dup + rep) case, we found very few colonies which were supposed to hold mini-chromosome and therefore showed Ura^+^ phenotype but which, by unknown reason, did not lose mini-chromosome and consequently showed 5-FOA^−^ phenotype. Two representatives of this type of colonies were marked with green-colored arrow

We cultivated the Chr2-6 (dup + rep) transformant and Chr11-2 (dup + rep) transformant in YPDA liquid medium overnight. Cells were then plated onto a YPDA plate and replica-plated on Ura minus, Leu minus, Leu minus and Ura minus media, as well as 5-FOA and YPDA plates as a control (Fig. 8). In this experiment, we do not have to hypothesize the occurrence of a suppressor mutation because there is no selection pressure, given the second copy of the target region is present on the mini-chromosome. Therefore, if the target region is essential for viability, we should not expect any colonies on 5-FOA medium. As shown in Fig. 8a, b, several types of colonies were obtained on 5-FOA plates along with other replica plates for both the Chr2-6 and Chr11-2 regions. Indeed, most of the transformants which showed growth in 5-FOA plate also grew on Ura-minus medium. Usually, Ura^+^ colonies would not grow on 5-FOA medium, but when cells are proliferating, we think that some Ura^+^ cells generate Ura^−^ cells by loosening mini-chromosome and those Ura^−^ cells can form colonies on 5-FOA medium. In fact, when we took cells from the colonies formed on 5-FOA plate and streaked on Ura minus medium, we found that those cells did not show any growth (data not shown). Thus, all of these observations suggest that specific suppressor mutation might not be responsible for viability in the case of replacement of Chr2-6 and Chr11-2 regions. Therefore, there is still apparent inconsistency is present between this study and previous study.

### Analyzing essentiality of chromosomal regions

**Fig. 9:**
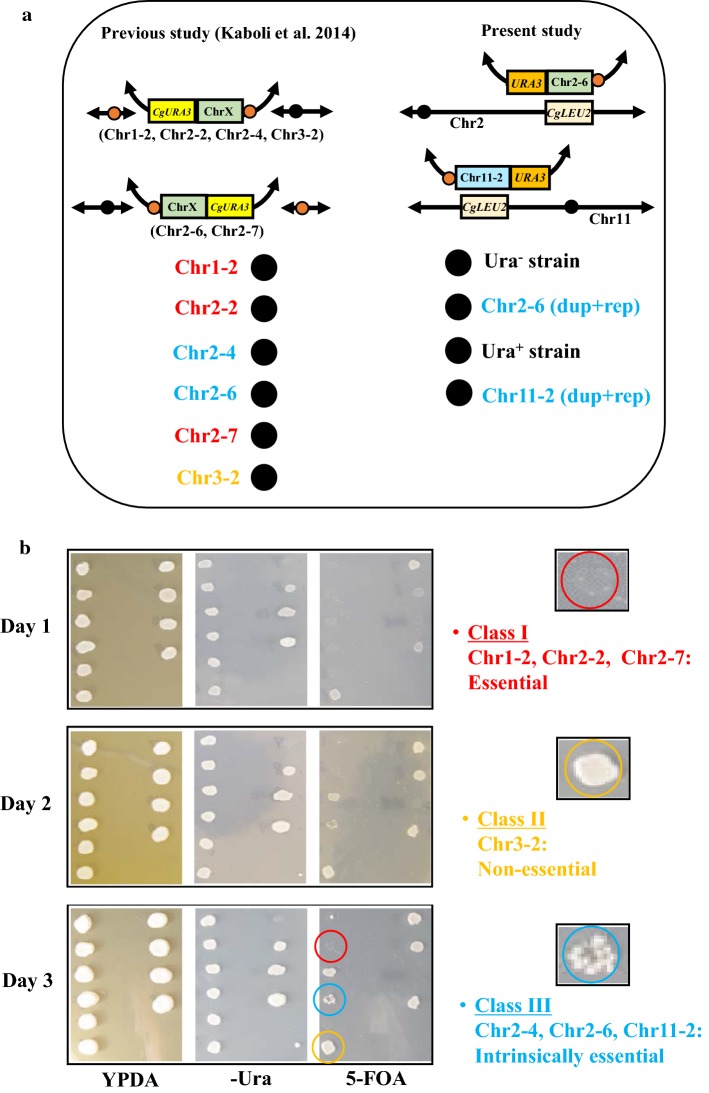
Spot assay of mini-chromosome harboring strains. **a** Represents position of spots in YPDA, -Ura and 5-FOA plate. Left side of **a** indicates the structure of chromosome and spots of transformants harboring Chr1-2, Chr2-2, Chr2-4, Chr2-6, Chr2-7 and Chr3-2 regions in mini-chromosome constructed by previous study (Kaboli et al. 2014). Right side of **a** indicates the structure of chromosome and spots of Chr2-6 (dup + rep) and Chr11-2 (dup + rep) transformants. Spots of Ura^−^ and Ura^+^ strains were negative and positive control, respectively. **b** Represents the spotting assay of all strains in YPDA, -Ura and 5-FOA plate from day 1 to day 3. Three types of colonies were found according to the growth of transformants and these transformants were categorized as Class I, Class II and Class III. Transformants harboring Chr1-2, Chr2-2 and Chr2-7 regions belong to Class I and regions deleted in Class I transformants were considered to be essential while transformants harboring Chr3-2 region belong to Class II and the region deleted in Class II transformants was treated as non-essential. The third type of transformants harboring Chr2-4, Chr2-6 and Chr11-2 regions belong to Class III and regions deleted in Class III transformants were considered to be intrinsically essential. From each class of transformants, one representative colony was circled. Colony representing Class I transformants (harboring Chr2-2 region) was circled by red color, Class II transformants (harboring Chr3-2 region) was circled by yellow color and Class III transformants (harboring Chr2-6 region) was circled by blue color

To solve the apparent discrepancy, we again chose Chr2-6 (dup + rep) and Chr11-2 (dup + rep) transformants along with some of the transformants constructed in previous study (Kaboli et al. 2014) harboring Chr1-2, Chr2-2, Chr2-4, Chr2-6, Chr2-7 and Chr3-2 regions in the mini-chromosome. We cultivated all of those transformants in YPDA liquid medium overnight. Cells were then spotted onto YPDA, Ura minus and 5-FOA plate (Fig. 9). We incubated those plates and each day we observed the growth of colonies and took the photos as shown in Fig. 9. We found 3 kinds of phenotypes on the colonies originated from those transformants. We categorized those transformants as Class I, Class II and Class III. Class I transformants (harboring Chr1-2, Chr2-2 and Chr2-7 regions) did not show growth even after a long period of incubation in 5-FOA medium. Thus, the target region (Chr1-2, Chr2-2 and Chr2-7) is considered to be essential for viability. Class II transformants (harboring Chr3-2 region) showed regular growth in 5-FOA medium even after day 1. Therefore, the chromosomal region (Chr3-2) that was deleted from this transformants is considered to be non-essential. On the other hand, Class III transformants (harboring Chr2-4, Chr2-6 and Chr11-2 regions) did not show growth after day 1, but they gradually formed so called papillae colonies in 5-FOA medium within day 3. From these observations, we defined that the chromosomal region (Chr2-4, Chr2-6 and Chr11-2 regions) deleted from Class III transformants is intrinsically essential but lethality could be compensatable, and consequently adaptable cells appeared during a longer incubation. We will discuss this interesting issue in Discussion section.

## Discussion

There is an inconsistency between the results of this study and a previous study where mini-chromosomes comprising target regions were constructed by PCS followed by mini-chromosome loss assays, which led to lethality. However, in this study, direct deletion through replacement of the target region was not found to be lethal. When we split the chromosome at any chromosomal site by PCS, we thought that telomere repression may happen to the region close to the artificial telomere. Therefore, if expression of an essential gene becomes repressed by telomere repression, the cell would die. In order to avoid telomere repression occurring for an essential gene, Kaboli et al. (2014) split the chromosome at least 1 kb from the essential genes. After splitting both edges of a particular target region to create a mini-chromosome, comprising the target region along with marker and confirming that transformants containing mini-chromosome are viable, a mini-chromosome loss assay was conducted. Because transformants containing the mini-chromosome are viable before mini-chromosome loss, we thought that telomere repression does not occur. Thus, a simple explanation for lethality after mini-chromosome loss is that the target region may have a gene-pair that results in synthetic lethality as the target region contains only non-essential genes.

Here, we deleted the same chromosomal region by a one-step replacement. Thus, we can assume that telomere repression does not occur because the chromosome is continuous and not split. Moreover, in this case there is no newly added artificial telomeres in the resultant chromosome. In this way, expression of essential genes in the left and right side close (i.e. within 1 kb) to the replaced region is not be repressed and should remain functional. However, the target regions had been deleted by replacement with the *CgLEU2* marker and further mini-chromosome loss assays of the *CgLEU2* marker also resulted in viable cells. Thus, we need to explain how the resulting transformed cells could be viable by one-step replacement with a marker gene.

We thought that during selection of transformants, suppressor mutations might occur that suppress the lethality caused by deletion of the target region. However, we confirmed that suppressor mutation was not responsible for viability, rather loss of essential regions (Chr2-6, Chr11-2) could be occasionally compensatable (in Class III transformants). We reasoned that possible gross alteration of gene expression caused by deletion of many genes at a time may affect physiological change, resulting in compensation or adaptation for viability. Therefore, the chromosomal regions which were deleted from these transformants could be considered as “intrinsically essential” regions. This idea is consistent with the “Mass action of gene” hypothesis (Bonney et al. 2015) for gaining adaptability. Mass action of gene hypothesis was reported as an idea that growth fitness is determined by gross change of gene expression caused by deletion of many genes at a time but not attributed to specific change of gene expression caused by the deletion of few critical genes. From these overall discussion, we came to a conclusion that change of entire gene expression profile may lead those cells to be viable.

Our study highlights an important caveat to evaluate whether a particular region of the *S. cerevisiae* genome is essential or non-essential or intrinsically essential for cell viability. We believe that prudent approaches such as replacement, splitting and mini-chromosome loss assay with careful observation of growth phenotype are needed for the analysis of essentiality or non-essentiality of a particular chromosomal region to understand precisely genome function in *S. cerevisiae.*

## Supplementary information

**Additional file 1: Table S1.** Primers used to generate DNA modules for replacement, splitting and duplication. **Table S2.** Primers used for colony PCR.

**Additional file 2: Fig. S1.** Colony PCR analysis of replaced sub-regions of Chr2-2 region. **Fig. S2.** Colony PCR analysis of replaced sub-regions of Chr9-2 region. **Fig. S3.** Colony PCR analysis of replaced sub-regions of Chr11-2 region.

## Data Availability

The datasets generated during and/or analyzed during the current study are available from the corresponding author on reasonable request.
