# Supplementary material for: Systematic approach for assessing whether undeletable chromosomal regions in Saccharomyces cerevisiae are required for cell viability
Source: AMB Express. 2020 Apr 15;10:73. doi: 10.1186/s13568-020-01001-x (PMC7158983; doi:10.1186/s13568-020-01001-x)
Supplement: Supplementary file 1 — Additional file 1: Table S1. Primers used to generate DNA modules for replacement, splitting and duplication. Table S2. Primers used for colony PCR. [file 13568_2020_1001_MOESM1_ESM.docx]

**Fig. S1. Colony PCR analysis of replaced sub-regions of Chr2-2 region.** Each lane represents checking of left or right edge replacement of Chr2-2 sub-regions in independent transformants (T1, T2, T3, T4, T5 and T6). 1 kb band was the expected band for replacement of either left or right edge of sub-regions. (A) SJP 217 and SJP 119 were used for checking left edge whereas SJP 127 and SJP 382 were used for checking right edge replacement of A1+A2 sub-regions, respectively. (B) SJP 218 and SJP 119 were used for checking left edge whereas SJP 127 and SJP 383 were used for checking right edge replacement of B1+B2 sub-regions, respectively. (C) SJP 217 and SJP 119 were used for checking left edge whereas SJP 127 and SJP 456 were used for checking right edge replacement of A1+A2+B1 sub-regions, respectively. (D) SJP 426 and SJP 119 were used for checking left edge whereas SJP 127 and SJP 383 were used for checking right edge replacement of A2+B1+B2 sub-regions, respectively. (E) SJP 217 and SJP 119 were used for checking left edge whereas SJP 127 and SJP 478 were used for checking right edge replacement of A1+A2+B1 (Ex) sub-regions, respectively. (F) SJP 482 and SJP 119 were used for checking left edge whereas SJP 127 and SJP 383 were used for checking right edge replacement of A2 (Ex)+B1+B2 sub-regions, respectively.

**Fig. S2. Colony PCR analysis of replaced sub-regions of Chr9-2 region.** Each lane represents checking of left or right edge replacement of Chr9-2 sub-regions in independent transformants (T1, T2, T3, T4, T5 and T6). 1 kb band was the expected band for replacement of either left or right edge of sub-regions. (A) SJP 215 and SJP 119 were used for checking left edge whereas SJP 127 and SJP 368 were used for checking right edge replacement of A1+A2 sub-regions, respectively. (B) SJP 216 and SJP 119 were used for checking left edge whereas SJP 127 and SJP 369 were used for checking right edge replacement of B1+B2 sub-regions, respectively. (C) SJP 215 and SJP 119 were used for checking left edge whereas SJP 127 and SJP 458 were used for checking right edge replacement of A1+A2+B1 sub-regions, respectively. (D) SJP 428 and SJP 119 were used for checking left edge whereas SJP 127 and SJP 369 were used for checking right edge replacement of A2+B1+B2 sub-regions, respectively. (E) SJP 215 and SJP 119 were used for checking left edge whereas SJP 127 and SJP 480 were used for checking right edge replacement of A1+A2+B1 (Ex) sub-regions, respectively. (F) SJP 484 and SJP 119 were used for checking left edge whereas SJP 127 and SJP 369 were used for checking right edge replacement of A2 (Ex) +B1+B2 sub-regions, respectively.

**Fig. S3. Colony PCR analysis of replaced sub-regions of Chr11-2 region.**  Each lane represents checking of left or right edge replacement of Chr11-2 sub-regions in independent transformants (T1, T2, T3, T4, T5 and T6). 1 kb band was the expected band for the replacement of either left or right edge of sub-regions. (A) SJP 219 and SJP 119 were used for checking left edge whereas SJP 127 and SJP 386 were used for checking right edge replacement of A1+A2 sub-regions, respectively. (B) SJP 220 and SJP 119 were used for checking left edge whereas SJP 127 and SJP 387 were used for checking right edge replacement of B1+B2 sub-regions, respectively. (C) SJP 219 and SJP 119 were used for checking left edge whereas SJP 127 and SJP 459 were used for checking right edge replacement of A1+A2+B1 sub-regions, respectively. (D) SJP 429 and SJP 119 were used for checking left edge whereas SJP 127 and SJP 387 were used for checking right edge replacement of A2+B1+B2 sub-regions, respectively. (E) SJP 219 and SJP 119 were used for checking left edge whereas SJP 127 and SJP 481 were used for checking right edge replacement of A1+A2+B1 (Ex) sub-regions, respectively. (F) SJP 485 and SJP 119 were used for checking left edge whereas SJP 127 and SJP 387 were used for checking right edge replacement of A2 (Ex)+B1+B2 sub-regions, respectively.

**Table S1. Primers used to generate DNA modules for replacement, splitting and duplication**

| Name of the Primer | Description  (Chromosome number and co-ordinates) | Nucleotide sequence (5’-3’) |
| --- | --- | --- |
| Replacement |  |  |
| SJP 90 | Chr2 (318700-318749) Fw | TAATAAACCTCTTTTCGTATTTTTATGGCTTTCTTTGTGGAACATTGGGGGGCCGCCAGCTGAAGCTTCG |
| SJP 91 | Chr2 (324854-324903) Rv | TATATAACTCCATTGATGCTGAAGCGATTCCAAATAAAGTTCCGAAATCCAGGCCACTAGTGGATCTGAT |
| SJP 92 | Chr2 (324806-324855) Fw | GATTTCGTTATGTCCACCAATGCTTAAAGTGACCGTATTTTGGAGGAAGGGGCCGCCAGCTGAAGCTTCG |
| SJP 93 | Chr2 (330960-331009) Rv | TACTCAAGATGAAAGGTGCACATACGATTGCAGTTGCCTCAACTGATGAAAGGCCACTAGTGGATCTGAT |
| SJP 197 | Chr9 (87800-87849) Fw | TTAAGGACATTCACGGACGCATCCCAGAAATGCTGTGATTATACGCAACGGGCCGCCAGCTGAAGCTTCG |
| SJP 198 | Chr9 (95051-95100) Rv | ACAATAACCTCTATGAATCCAGACACAACCAAATAAAGAAAACTGAAGGGAGGCCACTAGTGGATCTGAT |
| SJP 199 | Chr9 (95000-95049) Fw | AAATGAATTTTTAGAGTAGGAGAAGAAGGTTGAAGAAATGAACAATCGCGGGCCGCCAGCTGAAGCTTCG |
| SJP 200 | Chr9 (102250-102299) Rv | TAATAGTGTGTAAATTGTGCGTTCAATTAGCAAAGAAAGGCTTGGAGAGAAGGCCACTAGTGGATCTGAT |
| SJP 201 | Chr2 (21816-21865) Fw | AGTGAATAATTTTAGATTTTGTTACATATAATTCTGCTTGCCTATCTCTTGGCCGCCAGCTGAAGCTTCG |
| SJP 202 | Chr2 (29607-29656) Rv | TTTTATTCCAACAATTATATGTGCTTGTATTCAGCTCTTTATTGAGTTTGAGGCCACTAGTGGATCTGAT |
| SJP 203 | Chr2 (29556-29605) Fw | TTCAATCACGTAAGGTGGAAGAGAATGACATGAAGATTGAGAAACAGTGAGGCCGCCAGCTGAAGCTTCG |
| SJP 204 | Chr2 (37347-37396) Rv | GAACCGAAAAGAACGATACCGACTTGACCAGGCTCCAAGTTCAAAGCCATAGGCCACTAGTGGATCTGAT |
| SJP 205 | Chr11 (188384-188433) Fw | ACATAAAGATAAACCAGTTTTTTTTGTTCAACGTCAATTGTGGCAATGTTGGCCGCCAGCTGAAGCTTCG |
| SJP 206 | Chr11 (196596-196645) Rv | GCTTATATGACTCCTTATAAAGACACAAGAAATACGGTGCCTGTTGCAGCAGGCCACTAGTGGATCTGAT |
| SJP 207 | Chr11 (196545-196594) Fw | TTGTCGATTTGGCTTGATTTCTGATTTGTAACGTCATTCACTGCCCCTGTGGCCGCCAGCTGAAGCTTCG |
| SJP 208 | Chr11 (204756-204805) Rv | ATAGTTTTGATCGAAGCTTCCTTTTCAGGGTTACGCCTATGGTAGATAGCAGGCCACTAGTGGATCTGAT |
| SJP 335 | Chr9 (98651-98700) Rv | ACTTCCGTTGGTTGGAAATACGAAGATGTTGTTGCCAAATTGGAAGCAAAAGGCCACTAGTGGATCTGAT |
| SJP 397 | Chr2 (33477-33526) Rv | TAATGATCTTTTCTTACTTTTGGTAGTAGTCAGAACATTAGAATCATCCAAGGCCACTAGTGGATCTGAT |
| SJP 399 | Chr2 (327909-327958) Rv | ACAAGATATTTGGTTGAATATGGGTCAGCTATCTTCTATCTGTCGTTACCAGGCCACTAGTGGATCTGAT |
| SJP 401 | Chr11 (200676-200725) Rv | CTCAAGCTAGGTGAGAACATACATTTGAGTATAAAATAAAAAATGCATATAGGCCACTAGTGGATCTGAT |
| SJP 416 | Chr2 (25687-25736) Fw | TATGGGCGTATCGATTAGAACCTCATAATGTCTTAATTTGGACGGACACTGGCCGCCAGCTGAAGCTTCG |
| SJP 417 | Chr2 (321752-321801) Fw | ATTATTTAATAATACTTTTAGCACAATAACGATTTACCATAAACTAAAGTGGCCGCCAGCTGAAGCTTCG |
| SJP 418 | Chr9 (91401-91450) Fw | GGTTTGACGCTTCTCGCATGAAATGTTTTAGTATCTTCAAATACACTTTCGGCCGCCAGCTGAAGCTTCG |
| SJP 419 | Chr11 (192465-192514) Fw | TATCAATAACGTTGCATTAGTTTCAGAGTCTTTATTTATTTCATTGAACCGGCCGCCAGCTGAAGCTTCG |
| SJP 460 | Chr2 (36037-36086) Rv | CTCAGACAATACTGAAGCTGTGTTAAAGACCTATTAGTTGAACATGTTATAGGCCACTAGTGGATCTGAT |
| SJP 461 | Chr2 (329134-329183) Rv | TCCGATTATGAAAGTGATAACGAATACAGAAATATGGATGAGGATTCAATAGGCCACTAGTGGATCTGAT |
| SJP 462 | Chr9 (100182-100231) Rv | TTATCTATGAATAAAATAAACGCCCAAAGAGGCACTGAAGACGCTGTGACAGGCCACTAGTGGATCTGAT |
| SJP 463 | Chr11 (201329-201378) Rv | CCAATGAGAAGATGTCTCGAAACATTCATTGAGTCGTGGACACCAGTGTTAGGCCACTAGTGGATCTGAT |
| SJP 464 | Chr2 (24423-24472) Fw | CAAGAAAGTTTGGTTTACTATGGACAATGGGGTCCCTACTATTTGTTCTTGGCCGCCAGCTGAAGCTTCG |
| SJP 465 | Chr2 (321014-321063) Fw | TCCTCAGTTATGCGCTCAGGTGACTTTCCAGCAAGTGAGCCGGCGCCCCTGGCCGCCAGCTGAAGCTTCG |
| SJP 466 | Chr9 (89474-89523) Fw | TTATTAGATCTCAAGTTATTGGAGTCTTCAGCCAATTGCTTTGTATCAGAGGCCGCCAGCTGAAGCTTCG |
| SJP 467 | Chr11 (190284-190333) Fw | TTGTAGTCAACGGCTTCTTAAGATCTTTGGCCTTGAGTTCAGCTATAAATGGCCGCCAGCTGAAGCTTCG |
| Splitting |  |  |
| SJP 13 | CA Primer | CCCCAACCCCAACCCCAACCCCAACCCCAACCCCAAAGGCCACTAGTGATCTGAT |
| SJP 519 | *CgLEU2* (1-50) Rv | CAAGATAGGGATGATTACAGAGCACACATTTCCGGGAAACACAGAATTGGGGCCGCCAGC TGAAGCTTCG |
| SJP 520 | *CgLEU2* (1636-1685) Fw | GCTATATTAGCTTGTGCATTCGCATGTATCGGCAAACGAACTTTACGTAAGGCCGCCAGCTGAAGCTTCG |
| SJP 522 | Chr2 (318699-318748) Fw | CTAATAAACCTCTTTTCGTATTTTTATGGCTTTCTTTGTGGAACATTGGGGGCCGCCAGCTGAAGCTTCG |
| SJP 523 | Chr2 (330961-331010) Rv | CTACTCAAGATGAAAGGTGCACATACGATTGCAGTTGCCTCAACTGATGAGGCCGCCAGCTGAAGCTTCG |
| Duplication |  |  |
| SJP 671 | Chr2 (318749-318798) Rv | TGAACCAGCGGAGTGCCTTTAGTATTATAGTTTAAAAAAGCTGGAATAGCGGCCGCCAGCTGAAGCTTCG |
| SJP 672 | Chr2 (330911-330960) Fw | AAGCGTTGATCAAGTATTCGGCGCCGTATTCCTTCGCTATTTTAAGCTTTGGCCGCCAGCTGAAGCTTCG |
| SJP 675 | Chr11 (188434-188483) Rv | AAATGACGTTGGGAAAAGATGTCTCTTCGCTGTTCCCAGACGTCTTGAAAGGCCGCCAGCTGAAGCTTCG |
| SJP 676 | Chr11 (204706-204755) Fw | TCAGAGAAAAGGACGGTCTATGGGCCATTATTGCTTGGTTAAATATCTTGGGCCGCCAGCTGAAGCTTCG |

**Table S2. Primers used for colony PCR**

| Name of the Primer | Chromosome number (coordinates) | Nucleotide sequence (5’-3’) |
| --- | --- | --- |
| SJP 118 | Chr2 (318549-318573) Fw | TTAGTTTACACCCGTCCCATGGCCGA |
| SJP 119 | *CgLEU2* (776-800) Rv | CCCACTAGTTCTCTAACAACGACGA |
| SJP 121 | *CNE1* (211-230) Fw | TCACAGGGTCGATTGCAAGG |
| SJP 127 | *CgLEU2* (776-800) Fw | TCGTCGTTGTTAGAGAACTAGTGGG |
| SJP 215 | Chr9 (87646-87670) Fw | CCCACAACAATGTCAACTTCATCTT |
| SJP 216 | Chr9 (94846-94870) Fw | CATATTCACATGTTTCTCATTTTTT |
| SJP 217 | Chr2 (21686-21710) Fw | ATAATACTAATGCATTTAAATCATA |
| SJP 218 | Chr2 (29426-29450) Fw | ATGATATATAAACAACTTCAATAAA |
| SJP 219 | Chr11 (188256-188280) Fw | CAACTCTTATCATTGACATCGTTCT |
| SJP 220 | Chr11 (196416-196440) Fw | TTTGCTCTTGCTGCCAATGCAGAAG |
| SJP 242 | *CNE1* (880-861) Rv | CTGGTGGTTCAGTGCCATCT |
| SJP 368 | Chr9 (95116-95140) Rv | CACAAACTCGAATCCAAGTTCAAAA |
| SJP 369 | Chr9 (102315-102339) Rv | TTAGATGAATACCGGCTCTATAGAA |
| SJP 382 | Chr2 (29672-29696) Rv | TACTAGTAACGTAAATACTAGTTAG |
| SJP 383 | Chr2 (37412-37436) Rv | GGTTCTCTTGACCAATTCACCTTCT |
| SJP 384 | Chr2 (29672-29696) Rv | AGTATCGAATCCATAAAAGCGACCA |
| SJP 385 | Chr2 (331026-331050) Rv | TTTTGCCGCAGCGGGTGGTGTGGGA |
| SJP 386 | Chr11 (196661-196685) Rv | AAAGCTTCTCGATGGAAGCAAAGAA |
| SJP 387 | Chr11 (204821-204845) Rv | GAAGAAAGTACGGCCATACTCGTTC |
| SJP 390 | Chr2 (324656-324680) Fw | GAAATTGGGTTCATTTGCTTTCAGT |
| SJP 411 | *CgHIS3* (401-425) Rv | CGCCTCCTTGAACGCTTGGCCCAGC |
| SJP 426 | Chr2 (25537-25561) Fw | TTCTTCAAAAGTTGGCGGAGGTGGA |
| SJP 427 | Chr2 (321602-321626) Fw | TGCAGCATCAGCTTATTGACCTCGC |
| SJP 428 | Chr9 (91251-91275) Fw | TGTTGAGTCAATTTTGTTTGCGTTT |
| SJP 429 | Chr11 (192315-192339) Fw | GTAGTCAGGTTTGGATTTACCAATA |
| SJP 456 | Chr2 (33542-33566) Rv | ATGTGTCTCGGGATACCTCAATTTC |
| SJP 457 | Chr2 (327974-327998) Rv | CGCCTGTGCAATTTTTTGCCTATCA |
| SJP 458 | Chr9 (98716-98740) Rv | GAAGCCAGGTAGAAAGTACACCACC |
| SJP 459 | Chr11 (200741-200765) Rv | TTTGAGAAATGGTTGAACCTTTCAC |
| SJP 478 | Chr2 (36102-36126) Rv | TGTGATTGCGCCTATTGCAGAAGGA |
| SJP 479 | Chr2 (329199-329223) Rv | AAAAGTAGATTTTCCCTCTAACAAA |
| SJP 480 | Chr9 (100247-100271) Rv | CGAAGAACTCAGTGCCATAACGGTG |
| SJP 481 | Chr11 (201394-201418) Rv | AAAGCAATTAGGTATGCTACCTCAT |
| SJP 482 | Chr2 (24273-24297) Fw | TATCTAGACAGGACTTGGTGCAAGA |
| SJP 483 | Chr2 (320864-320888) Fw | AATGGCTTTTTGCCTATTTTGGCAG |
| SJP 484 | Chr9 (89324-89348) Fw | TCTCTTTCTTCTTCCAAAGCAACGA |
| SJP 485 | Chr11 (190134-190158) Fw | AATCTGACAAGCCCTGAATGACATT |
| SJP 550 | *URA3* (116545-116569) Rv | GCTTCAAACCGCTAACAATACCTGG |
| SJP 690 | Chr2 (319525-319549) Rv | CAAAAACCATTGAATTATAGTACCA |
| SJP 692 | Chr11 (203955-203979) Fw | GGTGGGCCAGCTCCAGAGAGTGTCA |
| SJP 694 | Chr2 (329960-329984) Fw | CAAAGTCACGCAAATCTAATGTATC |
| SJP 696 | Chr11 (189410-189434) Rv | TAGAACGACGTTTAAAGGTCCTAGT |
| SJP 697 | pUG6 (7-26) Rv | CGAAGCTTCAGCTGGCGGCC |
