# Supplementary material for: Systematic approach for assessing whether undeletable chromosomal regions in Saccharomyces cerevisiae are required for cell viability
Source: AMB Express. 2020 Apr 15;10:73. doi: 10.1186/s13568-020-01001-x (PMC7158983; doi:10.1186/s13568-020-01001-x)
Supplement: Supplementary file 2 — Additional file 2: Fig. S1. Colony PCR analysis of replaced sub-regions of Chr2-2 region. Fig. S2. Colony PCR analysis of replaced sub-regions of Chr9-2 region. Fig. S3. Colony PCR analysis of replaced sub-regions of Chr11-2 region. [file 13568_2020_1001_MOESM2_ESM.pptx]

## Slide 1
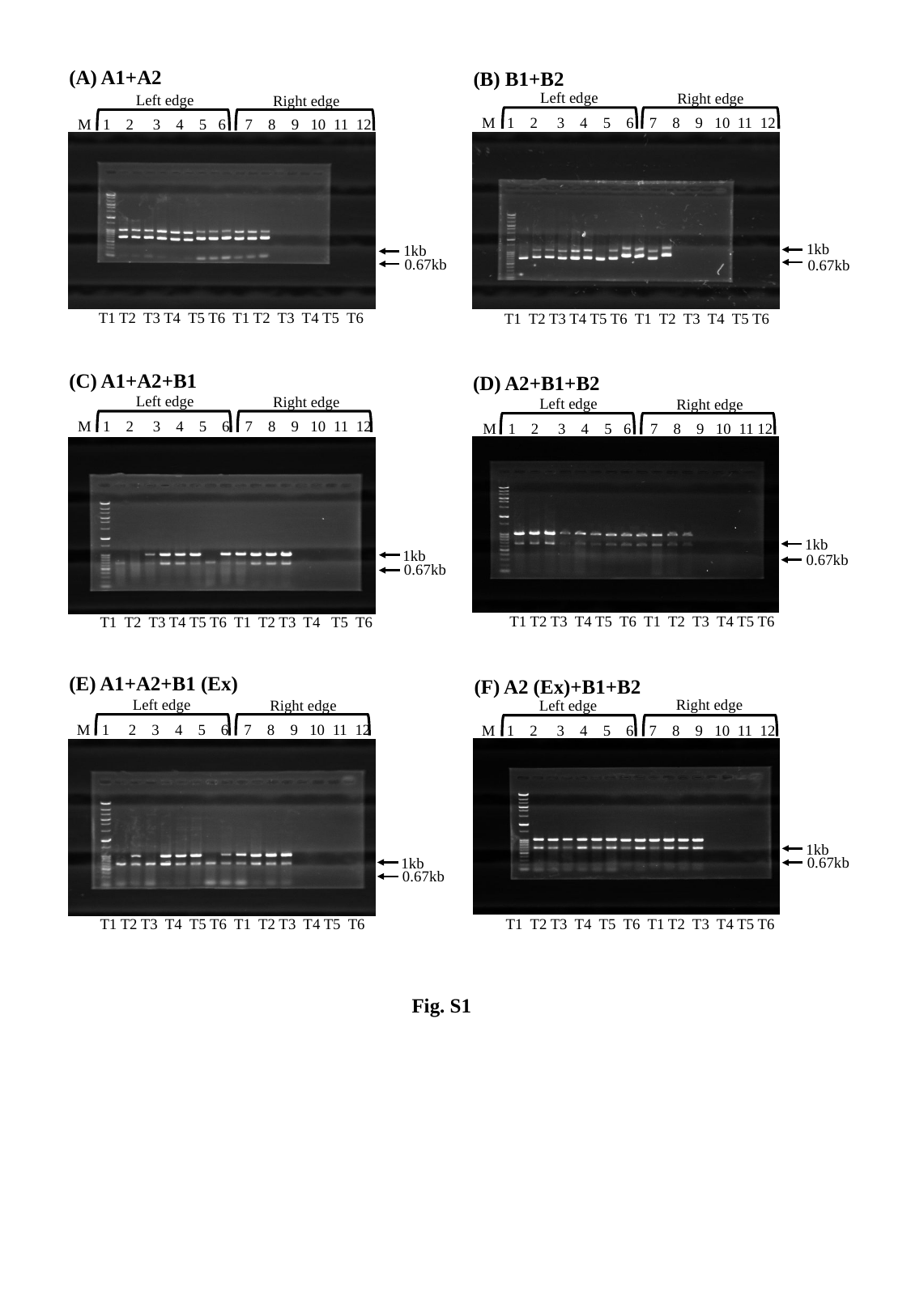

(A) A1+A2
(B) B1+B2
Left edge
Right edge
Left edge
Right edge
M 1 2 3 4 5 6 7 8 9 10 11 12
M 1 2 3 4 5 6 7 8 9 10 11 12
1kb
1kb
0.67kb
0.67kb
 T1 T2 T3 T4 T5 T6 T1 T2 T3 T4 T5 T6
 T1 T2 T3 T4 T5 T6 T1 T2 T3 T4 T5 T6
(C) A1+A2+B1
(D) A2+B1+B2
Left edge
Right edge
Left edge
Right edge
M 1 2 3 4 5 6 7 8 9 10 11 12
M 1 2 3 4 5 6 7 8 9 10 11 12
1kb
1kb
0.67kb
0.67kb
 T1 T2 T3 T4 T5 T6 T1 T2 T3 T4 T5 T6
 T1 T2 T3 T4 T5 T6 T1 T2 T3 T4 T5 T6
(E) A1+A2+B1 (Ex)
(F) A2 (Ex)+B1+B2
Left edge
Right edge
Right edge
Left edge
 M 1 2 3 4 5 6 7 8 9 10 11 12
 M 1 2 3 4 5 6 7 8 9 10 11 12
1kb
0.67kb
1kb
0.67kb
 T1 T2 T3 T4 T5 T6 T1 T2 T3 T4 T5 T6
 T1 T2 T3 T4 T5 T6 T1 T2 T3 T4 T5 T6
Fig. S1

## Slide 2
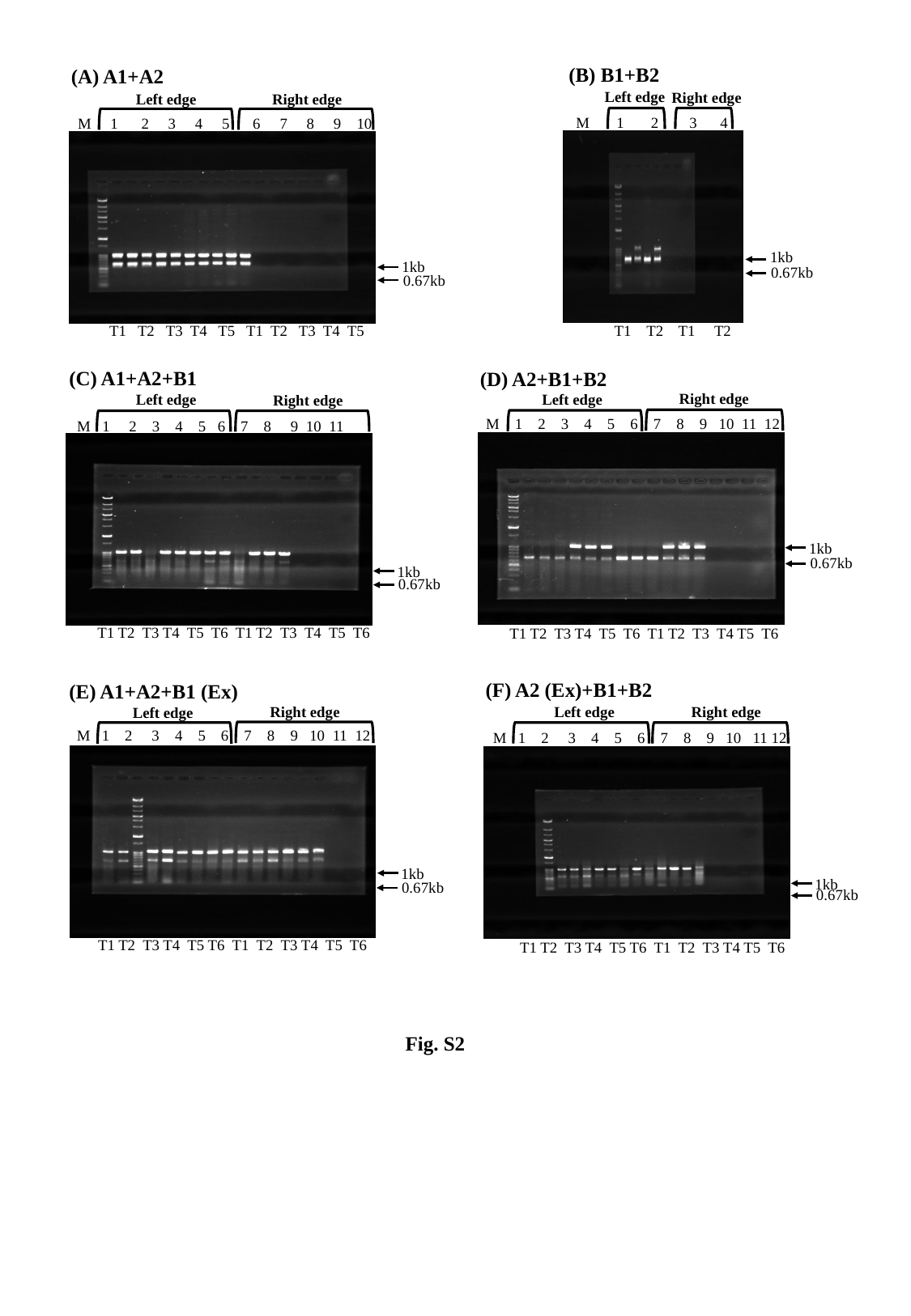

(B) B1+B2
(A) A1+A2
Left edge
Right edge
Right edge
Left edge
M 1 2 3 4
M 1 2 3 4 5 6 7 8 9 10
1kb
1kb
0.67kb
0.67kb
 T1 T2 T1 T2
 T1 T2 T3 T4 T5 T1 T2 T3 T4 T5
(C) A1+A2+B1
(D) A2+B1+B2
Right edge
Left edge
Left edge
Right edge
M 1 2 3 4 5 6 7 8 9 10 11 12
M 1 2 3 4 5 6 7 8 9 10 11 12
1kb
0.67kb
1kb
0.67kb
 T1 T2 T3 T4 T5 T6 T1 T2 T3 T4 T5 T6
 T1 T2 T3 T4 T5 T6 T1 T2 T3 T4 T5 T6
(F) A2 (Ex)+B1+B2
(E) A1+A2+B1 (Ex)
Left edge
Right edge
Right edge
Left edge
 M 1 2 3 4 5 6 7 8 9 10 11 12
 M 1 2 3 4 5 6 7 8 9 10 11 12
1kb
1kb
0.67kb
0.67kb
 T1 T2 T3 T4 T5 T6 T1 T2 T3 T4 T5 T6
 T1 T2 T3 T4 T5 T6 T1 T2 T3 T4 T5 T6
Fig. S2

## Slide 3
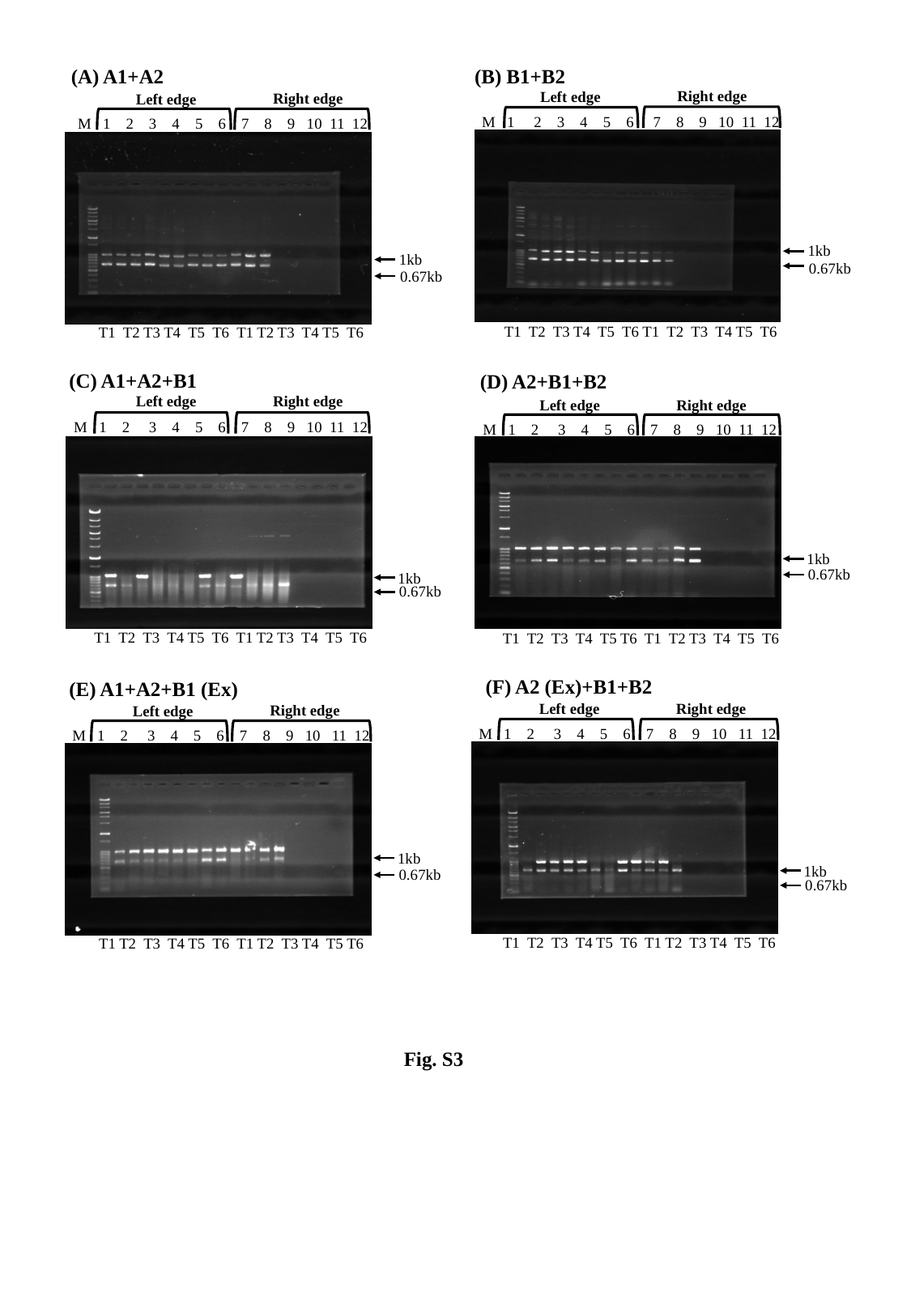

(B) B1+B2
(A) A1+A2
Right edge
Left edge
Right edge
Left edge
M 1 2 3 4 5 6 7 8 9 10 11 12
M 1 2 3 4 5 6 7 8 9 10 11 12
1kb
1kb
0.67kb
0.67kb
 T1 T2 T3 T4 T5 T6 T1 T2 T3 T4 T5 T6
 T1 T2 T3 T4 T5 T6 T1 T2 T3 T4 T5 T6
(C) A1+A2+B1
(D) A2+B1+B2
Left edge
Right edge
Left edge
Right edge
M 1 2 3 4 5 6 7 8 9 10 11 12
M 1 2 3 4 5 6 7 8 9 10 11 12
1kb
0.67kb
1kb
0.67kb
 T1 T2 T3 T4 T5 T6 T1 T2 T3 T4 T5 T6
 T1 T2 T3 T4 T5 T6 T1 T2 T3 T4 T5 T6
(F) A2 (Ex)+B1+B2
(E) A1+A2+B1 (Ex)
Right edge
Left edge
Right edge
Left edge
 M 1 2 3 4 5 6 7 8 9 10 11 12
 M 1 2 3 4 5 6 7 8 9 10 11 12
1kb
1kb
0.67kb
0.67kb
 T1 T2 T3 T4 T5 T6 T1 T2 T3 T4 T5 T6
 T1 T2 T3 T4 T5 T6 T1 T2 T3 T4 T5 T6
Fig. S3
